# Big data analysis of the impact of COVID-19 on digital game industrial sustainability in South Korea

**DOI:** 10.1371/journal.pone.0278467

**Published:** 2022-12-30

**Authors:** JuChan Na, Eun Joung Kim, JungYoon Kim

**Affiliations:** 1 Department of IT Convergence Engineering, Gachon University, Seongnam-si, Republic of Korea; 2 Culture Contents Technology Institute, Gachon University, Seongnam-si, Republic of Korea; 3 Department of Game Media, College of Future Industry, Gachon University, Seongnam-si, Gyeonggi-do, Republic of Korea; Sejong University, REPUBLIC OF KOREA

## Abstract

The COVID-19 pandemic has greatly influenced the lifestyle and entertainment activities of the society that has significantly increased the growth rate of the gaming industry. While the studies on the game industry, one of the leading content industries, related to the pandemic has been done from various perspectives, little attention has been taken in regards to how the pandemic have impacted on the national digital game production and its industrial sustainability as a whole. Thus, this study was conducted to analyze the changes in the domestic game industry before and after the COVID-19 outbreak using the big data analysis of semantic network. This study aims to understand the growing trend in the gaming industry that can be helpful for the marketing and production of future games, as well as to guide the establishment of the public game policies in the game industry. The results showed that the COVID-19 pandemic positively decreased the public’s worries and the government’s restrictions towards gaming due to game addiction as a mental disease. However, its sudden change in the gamer’s attitudes and the current gaming policies implied that for the sustainable development of the domestic game production, laws and regulations related to the game industry need to be reliable and planned on a long term basis since the industry is immensely large and is also related to several industries such as computing, programming, arts, and story contents. Accordingly, it is necessary to build an industrial ecology through which cluster complexes specializing in developing startups and small and medium-sized business can grow along with political support.

## Introduction

The prolonged Coronavirus pandemic (hereinafter referred to as ‘COVID-19’) since 2020 has brought enormous changes in people’s lifestyles. Face-to-face activities that form the basis of social life has converted into contact-free and outdoor leisure activities are also minimized [1]. These social changes have limited people from going outside, more work-from-home set-up, and people have more time for indoor entertainment such as playing video games, watching TV, and browsing the Internet. In the case of video games, the number of home users and their market has been greatly increasing since the COVID-19 outbreak. In South Korea, according to the *2021 White Paper on Korean Games* published by Korea Creative Content Agency (KCCA) specializing in games and content research, the Korean game market ranked fourth (6.9%) after the United States of America (21.9%), China (18.1%) and Japan (11.5%) [2]. The Korean video game market has been growing greatly in the aftermath of the COVID-19 outbreak. According to research on the actual condition of Korean game users in 2021 by KOCCA, the online game users rate in 2019 before the occurrence of COVID-19 stood at 65.7%, but in 2021, it rose to 71.3% [3], which is the greatest growth (5.6%) of all in five years since 2017. In addition to the number of total users, the users’ total play-time has also increased after the Coronavirus outbreak regardless of the device [4].

The studies with the focus on the online-gaming related to the COVID-19 pandemic have been done from various perspectives. Yoon et al. designed a serious game aiming to prevent the virus disease and studied how it could directly change the target user’s behaviors (especially the children) [5]. Kim and Kang analyzed why the Nintendo Switch game console gained a great popularity during the COVID-19 pandemic in South Korea [6]. Internationally, some research studied the direct effects of games on active players during the pandemic period [7] or investigated the changes in player population size and weekly patterns [8]. Bryl et al. examines the extent and level of the pandemic impact on video game by analyzing the emotional narration of articles related to Covid-19 effects on the game industry [9]. Such research has been focusing on how the pandemic situation has influenced gaming but little attention has been taken regarding the relationships between the social issues of the epidemic outbreak and the national game industry as a whole. As discussed, the game users have been increasing and it is expected that the size of its game market will also be increasing.

Thus, this study was conducted to identify and makes a comparison of the social issues associated with the Korean game industry between 2019 before COVID-19 pandemic and 2020 after its outbreak by using a big data analysis technique. This study has three main contributions as follows.

- The study can be helpful in understanding the trend of the Korean game industry.
- The results of the study can be helpful in establishing public game policies for the better improvement of the entire game industry in South Korea.
- The results of the study can also be helpful in predicting the upcoming trends in gaming contents production, distribution, and consumption which will be significantly useful for the marketing and game content industries.

The remainder of this paper is organized as follows. Section 2 provides a discussion on the relevant studies. The background of the study is discussed in Section 3. Section 4 outlines the research methods used in this study. The results and the discussion on their interpretations are provided in Section 5 and Section 6 concludes the study.

## Literature review

Due to the rapid progress of information and communication technologies, every information in our daily life is digitalized and massively produced. People as both consumer and producer obtain and process digitalized information and knowledge from online communities, read online newspapers through portal sites, and access video games via the Internet. The accumulated big data is so large, fast, and complex that it is difficult or impossible to process by using traditional analysis methods. Thus, big data are processed more efficiently using a Big Data Technology that is designed to analyze, process, and extract information from extremely complex and large data sets [10]. As an example of the big data analysis in the field of online games, Kang et al. collected the log data of the game *Battle Ground* and identified the users’ behavioral patterns in order to suggest the way in which users can be protected from playing abnormally [11]. D. H. Youm collected and analyzed users’ reviews from Google Play Store to propose a direction for each game genre [12].

There is another field of study about how the COVID-19 pandemic has made changes in people’s daily life in South Korea by using big data analysis. E. M. Kang studied certain changes in consumers’ fashion recognition before and after the pandemic [13]. J. M. Lee proposed market strategies for home fitness content whose market has been growing greatly after people’s self-restraint of outside activities [14]. Advanced IT infrastructure has been built in South Korea and big data analysis will be of great help to understand social issues and develop governmental policies [15].

The following related studies deals with the utilizing classification and prediction methods to understand the trends in the COVID-19 pandemic. In the study of Zhan et al. [16], a data-driven coding method has been utilized for predicting the COVID-19 spreading profile in any given population that shows an initial phase of epidemic progression. Based on the results of the study shows that the peak of infection cases in South Korea is before mid of April 2020 (0.01% of its population), end of March 2020 in Italy (0.5% of its population), and end of May 2020 in Iran (0.5% of its population). Guleria et al. [17] proposed Fine-tuned Ensemble Classification approach for predicting the death and cure rates of patients from the COVID-19 infection using Machine Learning techniques. The experimental results of the study yielded an F1-score of 94% as compared to other classifier techniques. The study of Sou Hyun Jang [18] attempted to analyze the multilevel factors associated with COVID-19 preventive practices in South Korea using an ordinary least squares (OLS) regression technique. The results showed that multilevel efforts are needed in promoting preventive behaviors.

Big data analytics can also be utilized to understand current events to support the preparation for the future events. In the study of Bag et al. [19], the Fuzzy Total Interpretive Structural Modeling (TISM) approach was used to categorized the multiple barriers in the humanitarian supply chain management, and to develop the contextual interrelationships. The approach was found effective to classify and understand the barriers that were helpful in the sustainable humanitarian supply chain management. In another study, Bag et al. [20] focused on identifying the reasons for which firms engaging in manufacturing activities adopt big data analytics-powered artificial intelligence. Their theoretical framework was statistically validated to provide insights regarding the role of institutional pressures on resources and their effects on the adoption of big data analytics-powered artificial intelligence.

Ali et al. [21] proposed a novel fuzzy ontology-based semantic knowledge with Word2vec model to improve the task of transportation features extraction and text classification using the Bi-directional Long Short-Term Memory (Bi-LSTM) approach. Their proposed method has showed satisfactory improvement in both the extraction of features and the classification of the unstructured text of social media. In their another study, Ali et al. [22], proposed an ontology and latent Dirichlet allocation (OLDA)-based topic modeling and word embedding approach for sentiment classification in order to retrieve transportation content from social networks, to remove the irrelevant contents to extract meaningful information, and to generate the topics and features from extracted data.

Most of the existing studies have been conducted in varying perspectives but limited studies were focused on how the COVID-19 pandemic has influenced the gaming perspectives of the society. In this regard, in order to understand the current trend of the gaming industry, this study was conducted aiming to help building the sustainability of the gaming industry in South Korea.

## Backgrounds

### Text mining

Text mining refers to an AI technology that transforms unstructured text data sets into normalized and structured data suitable for analysis [23]. Text mining allows the extraction of keywords and shows the frequency of those extracted keywords, indicating their importance [24].

### Semantic network analysis

**Fig 1:**
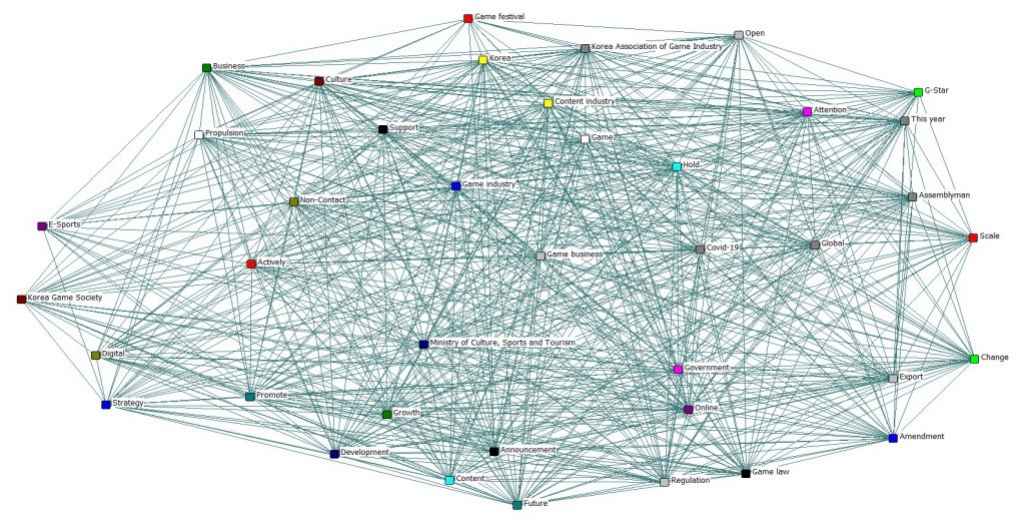
Overall network for game industry related keywords.

Semantic Network Analysis (SNA) is one of the social network analysis tools that refers to a text-based big data analysis [25]. SNA visualizes how information is connected with dots and lines as shown Fig 1, which allows us to figure out the characteristics of the overall network. SNA has an advantage to visualize the relational structure between social issues associated with a particular word while discovering what kind of role the word plays in its entire texts or with other words [26]. Among the various text mining techniques, SNA is to more objective and accurately measure the structural relationship between the particular word and the overall text [27]. Thus, SNA is widely used as a visual text analytics in the natural science fields such as physics and medical science as well as in social science including sociology, economics, and business administration through the various channels of big text data such as SNS or website news [28].

SNA extracts keywords that appear on the overall network and finds the relationships among these keywords (nodes) to analyze the detailed characteristics of the target information data [29].

### CONCOR

The ‘equivalence’ network shows whether the keywords or nodes within the network have similar levels of relationships with one another. When the keywords or nodes are in the same position, it is called a structural equivalence. The keywords or nodes within the structural equivalence can be substituted for each other.

**Fig 2:**
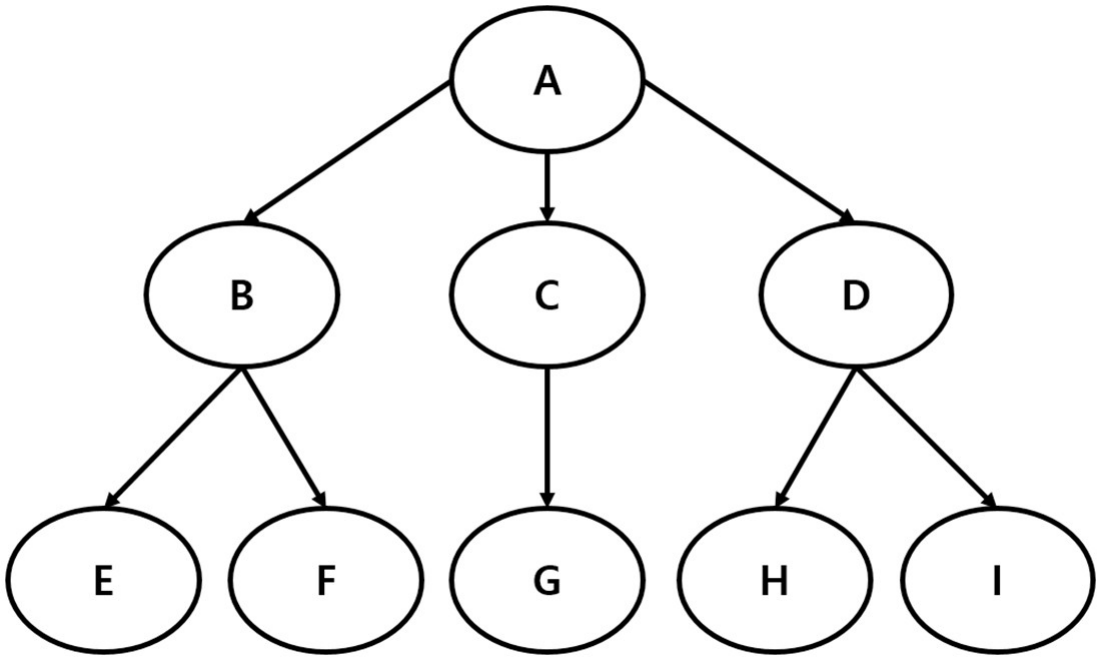
Keywords equivalence network.

As shown in Fig 2, the groups showing its structural equivalence are the keywords of both E and F and both H and I, which means that they can be substituted for each other [30, 31]. The structural equivalence analysis divides a large group of keywords into smaller-sized groups to allow us to easily understand the significance and meanings that the keywords intend to deliver [32]. For the structural equivalence analysis, the CONCOR (CONvergence of iterated CORrelations) procedure is the most widely used. CONCOR is used to group and scale keywords on the co-occurrence matrix based on the Pearson correlation coefficient [33].

### Co-occurrence matrix

**Fig 3:**
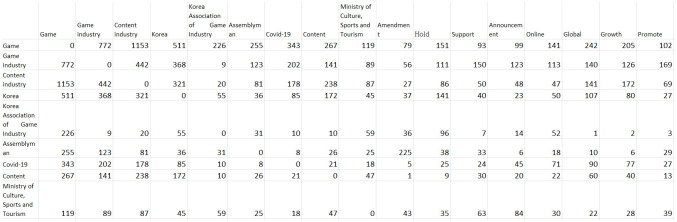
Extracted keywords co-occurrence matrix.

Before conducting the CONCOR procedure, the co-occurrence matrix among the keywords needs to be extracted. A co-occurrence matrix is a matrix that shows how many times keyword A in a row and keyword B in a column appear at the same time in the text. The co-occurrence matrix that was extracted in this study is illustrated in Fig 3.

Textom is a data processing program that collects text data through various acquisition channels such as news, blogs, and web documents and it also provides highly practical data that can be applied to various big data programs. It then refines the keywords, calculates the frequency, and extracts its co-occurrence matrix [34–38]. Textom is frequently used in studies for keyword analysis and as of March 2021, there are approximately 300 articles that were using Textom. Several studies utilized Textom to automatically collect a large amount of big data from portal sites and social media such as Twitter or Facebook [39]. Another method that this study has utilized is the UCIENT6 which can be used for various network analyses such as degree centrality and structural equivalence. UCIENT6 provides a NETDRAW function that can visualize the obtained results [40, 41].

## Research method

### Research process

**Fig 4:**
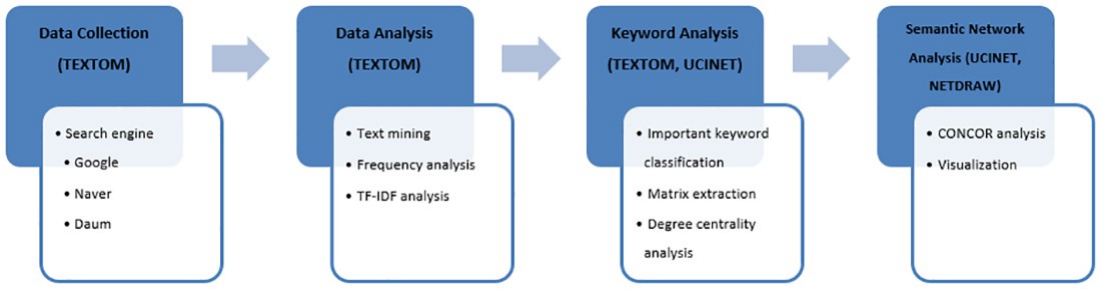
Research methodology block diagram.

This study utilized Textom in order to collect data and extract keywords. Then, SNA and its visualization were conducted through the UCINET6 program. Fig 4 summarizes the entire process with the used programs for this study.

The research methodology procedures are depicted in Table 1.

**Table 1:** The methodology processes and their descriptions.

Category		Description	
Preparation step	Data collection	◎ Collection keyword	
		· Game industry	
		◎ Period of collection	
		· Jan. 1, 2019 ~ Dec. 31, 2019(1 year)	
		· Jan. 1, 2020 ~ Dec. 31. 2020(1 year)	
		◎ News data collection	
		· Program: TEXTOM	
		· Search engine: Google(www.google.com)	
		Naver(www.naver.com)	
		Daum(www.daum.net)	
		Year of 2019	Year of 2020
		• Google: 136 • Naver: 710 • Daum: 763 • Total number: 1,609	• Google: 174 • Naver: 748 • Daum: 690 • Total number: 1,612
	Data analysis	◎ Refinement and extraction	
		· Program: TEXTOM	
		· Collected 1609 news data in 2019 and then extracted 6071 keywords	
		· Collected 1612 news data in 2020 and then extracted 5636 keywords	
		· Frequency analysis	
		· TF-IDF analysis	
Keyword analysis and visualization	Keyword analysis	◎ Keyword analysis	
		· Program: TEXTOM, UCINET	
		· Classified 40 important keywords	
		· Extracted Co-occurrence matrix	
		· Degree centrality analysis	
	Semantic network analysis	◎ Semantic network analysis	
		· Program: UCINET, NETDRAW	
		· CONCOR	
		· Visualization	
Conclusion			

### Data collection

**Fig 5:**
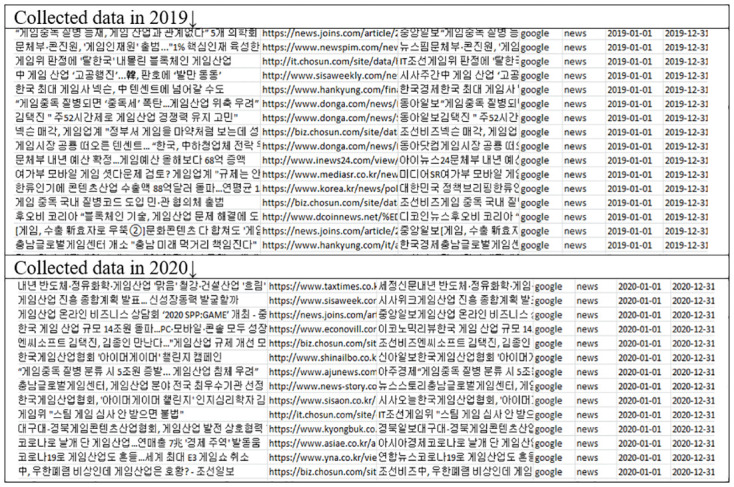
The collected data.

Data were collected from Google, Naver, and Daum which are the three leading search engines in South Korea. The search keyword was ‘game industry’. Data was collected separately, first, in the year 2019 before the spread of COVID-19 and then in the year 2020 as shown in Fig 5. In this study, data is purposely limited to news data only and unnecessary data were neglected [42]. For visualizing the keywords, this paper utilized the NETDRW program.

### Keyword extraction

Among the collected news data, unnecessary keywords for analysis such as name, company name, and area were removed. The top 40 words in frequency were selected and the TF-IDF value was calculated. TF-IDF refers to the importance of those keywords and their degree of centrality between keywords. TF-IDF is a statistical figure that shows the relative importance of a specific keyword of a document in a collection of documents [43]. The TF-IDF equation is illustrated in the Formula (1).


$$TF\times IN(\frac{D}{DF})$$ (1)

TF: Frequency of relevant keyword

IN: Natural logarithm

D: Total number of documents

DF: Number of documents which relevant words are included

The degree of centrality is the number of link incidents that shows how much a specific keyword is directly related to other keywords [44]. The degree of centrality was calculated by using UCINET6.

## Results

### Analysis of top 40 keywords before COVID-19 outbreak

**Fig 6:**
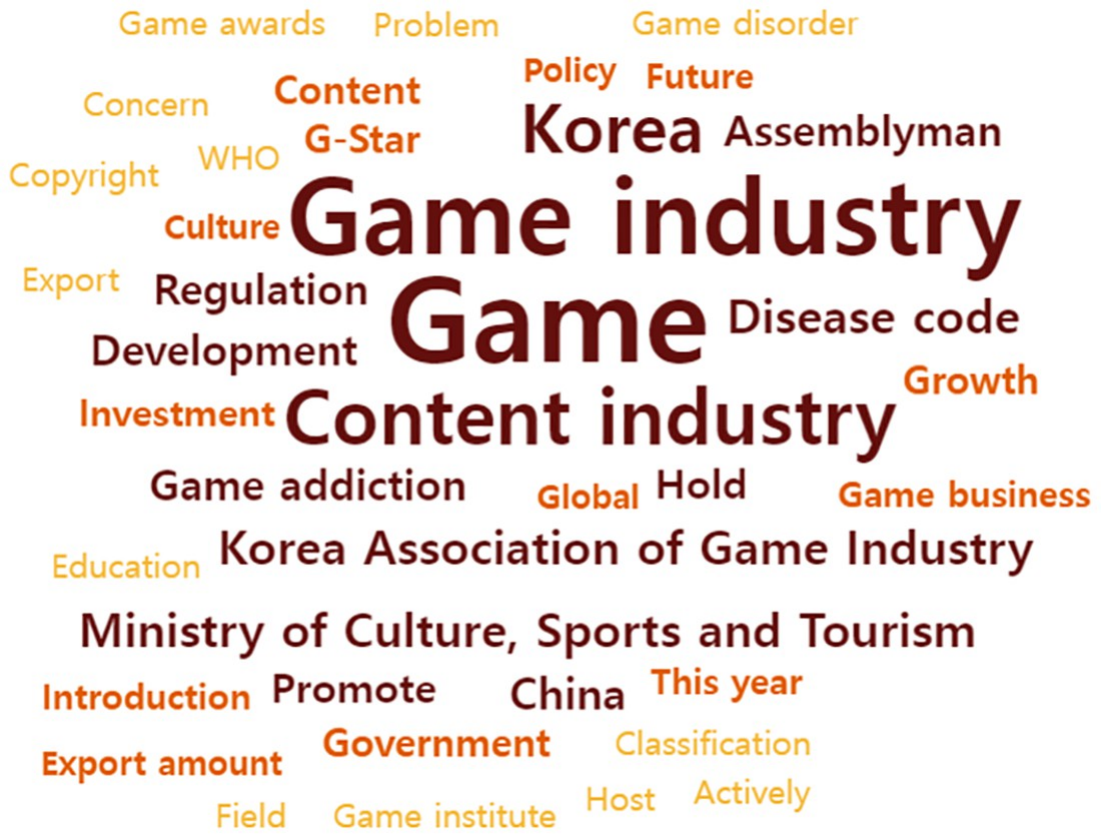
Word cloud of the top 40 keywords in 2019.

The top 40 keywords in frequency were selected and visualized in Fig 6.

The top 40 important keywords are as follows: Game (1195), Game industry (1032), Content industry (641), Korea (525), Disease code (258), Korea Association of Game Industry (255), Ministry of Culture, Sports and Tourism (254), China (209), Game addiction (184), Development (183), etc.

The TF-IDF is as follows: Game (1070), Content industry (792.942), Korea (780.262), Game industry (741.966), Disease code (556.666), Korea Association of Game Industry (543.429), Ministry of Culture, Sports and Tourism (532.156), China (516.379), Assemblyman (481.785), and Game addiction (479.181).

The degree of centrality is as follows; Game (0.147), Game industry (0.102), Content industry (0.091), Korea (0.069), Disease code (0.032), Korea Association of Game Industry (0.027), Ministry of Culture, Sports and Tourism (0.027), Game addiction (0.024), China (0.022), Regulation (0.021), etc.

In summary, it was found that the most important keywords were Game, Game industry, Content industry, and Disease code. The overall results are depicted in Table 2.

**Table 2:** The top 40 important keywords of the game industry in 2019.

Keyword	Freq	TF-IDF	Degree	Keyword	Freq	TF-IDF	Degree
Game	1195	1070.343	0.147	Introduction	103	309.138	0.013
Game industry	1032	741.966	0.102	This year	98	281.506	0.009
Content industry	641	792.942	0.091	Game business	96	281.186	0.012
Korea	525	780.262	0.069	Export amount	82	276.835	0.011
Disease code	258	556.666	0.032	Policy	80	265.834	0.01
Korea Association of Game Industry	255	543.429	0.027	Global	79	247.655	0.008
Ministry of Culture, Sports and Tourism	254	532.156	0.027	Culture	78	244.52	0.009
China	209	516.379	0.022	Export	77	262.807	0.01
Game addiction	184	479.181	0.024	WHO	77	235.053	0.01
Development	183	431.786	0.018	Copyright	76	278.905	0.011
Assemblyman	182	481.785	0.016	Education	72	247.113	0.007
Regulation	177	441.264	0.021	Game disorder	72	239.251	0.009
Promote	171	410.359	0.018	Game institute	71	243.681	0.008
Hold	158	401.191	0.019	Classification	68	221.424	0.008
G-Star	144	426.892	0.013	Game awards	66	240.616	0.012
Growth	139	364.346	0.016	Problem	66	224.029	0.007
Content	138	358.231	0.019	Field	62	207.1	0.007
Government	136	362.422	0.015	Concern	61	210.544	0.008
Investment	127	362.048	0.008	Host	60	197.341	0.009
Future	105	300.466	0.013	Actively	60	200.419	0.004

### CONCOR analysis of game industry in 2019

**Fig 7:**
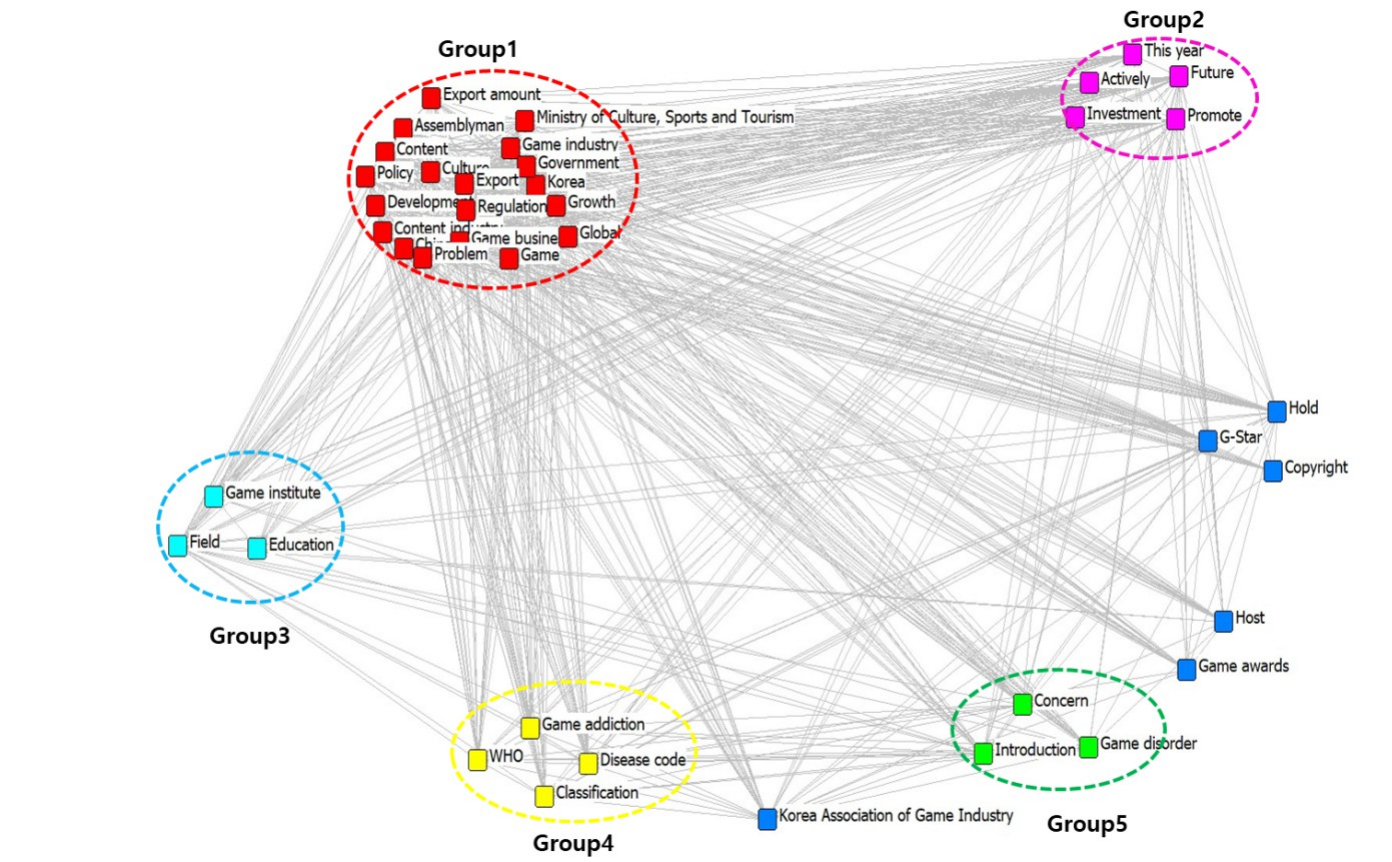
Visualization of CONCOR analysis in 2019.

By using the CONCOR and UCINET 6 programs, 40 keywords were selected for analysis as shown in Table 3 and its visualization is illustrated in Fig 7.

**Table 3:** Keyword groups based on CONCOR analysis in 2019.

Group	Group name	Keyword
Group 1 (red circle) (19)	Game industry	Game, Game industry, Content industry, Korea, Policy, Global, Ministry of Culture Sports and Tourism, China, Export amount, Development, Assemblyman, Regulation, Game business, Growth, Content, Government, Problem, Culture, Export
Group 2 (pink circle) (5)	Game industrial expansion	Investment, Promote, Future, Activity, This year
Group 3 (sky blue circle) (3)	Development of human resources	Education, Game institute, Field
Group 4 (yellow circle) (4)	Game addiction	WHO(World Health Organization), Game addiction, Disease code, Classification
Group 5 (green circle) (3)	Impediment to game industrial development	Introduction, Concern, Game disorder
-	Keywords which are excluded	Host, Game awards
-	Keywords which are excluded	Hold, G star, Copyright

The CONCOR analysis showed that Group 1 (red circle) includes Game, Game industry, Content industry, Korea, policy, and global, thus, it was named ‘Game industry’. Group 2 (pink circle) includes Investment and future, thus, it was named ‘Game industrial expansion’. Group 3 (sky blue circle) includes Education and Game institute, thus, it was named ‘Development of human resources’. Group 4 (yellow circle) includes World Health Organization (WHO) and Game addiction, thus, it was named ‘Game addiction’. Group 5 (green circle) includes Introduction and Concern, thus, it was named ‘Impediment to game industrial development’. Group 6 includes Host and Game awards while Group 7 includes Hold, G star, and Copyright which were excluded from the analysis because it was difficult to find out their meaning and significance. The details are depicted in Table 3.

The keywords can be classified into five groups that include Game industry, Game industrial expansion, Development of human resources, Game addiction, and Impediment to game industrial development. Thus, it can be suggested that the Korean game industry trend in 2019 was directed in two opposite ways, first, to a game industrial expansion associated with Game R&D and educating the game human resources, and second, to impediments to game industrial growth associated with game addiction or game disorder.

### Analysis of top 40 keywords after COVID-19 outbreak

**Fig 8:**
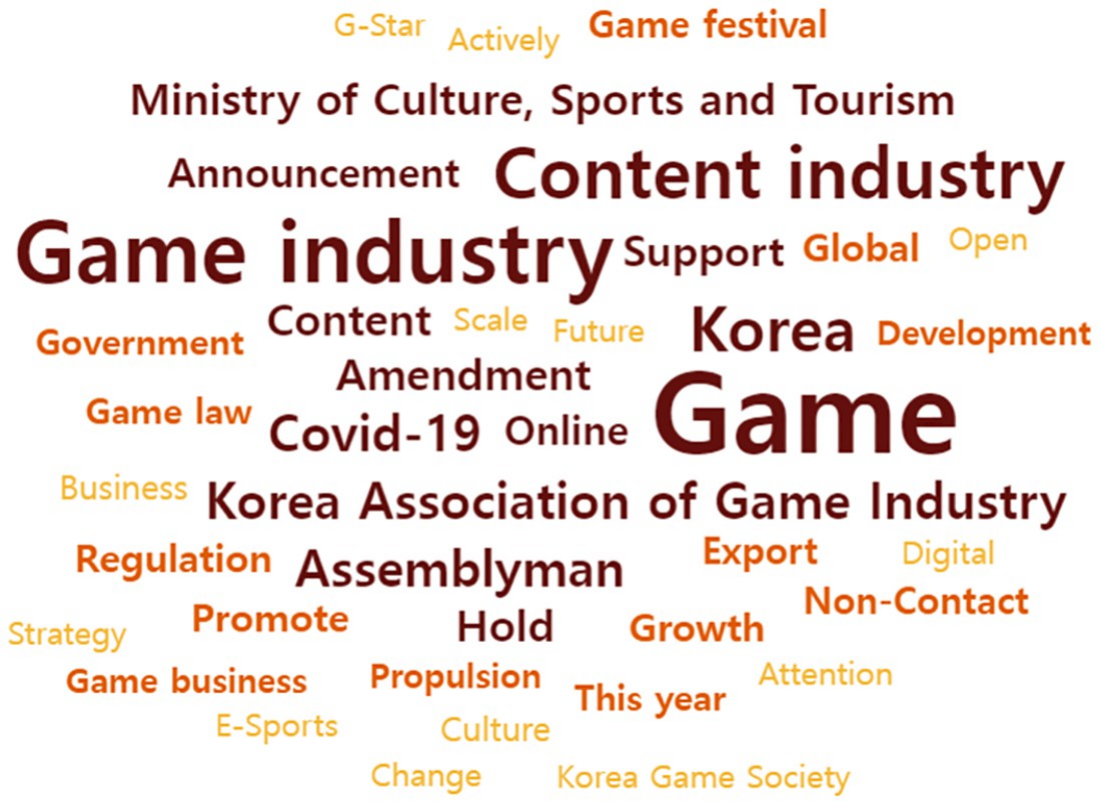
Word cloud of the top 40 keywords in 2020.

The top 40 important keywords were selected by collecting and analyzing news articles regarding the game industry in 2020 after the COVID-19 outbreak in South Korea. Its word cloud analysis is illustrated in Fig 8.

The results are Game (1232), Game industry (858), Content industry (639), Korea (509), Korea Association of Game Industry (344), Assemblyman (341), COVID-19 (332), Content (241), Ministry of Culture, Sports and Tourism (235), Amendment (232), etc.

The TF-IDF is as follows: Game (1021.64), Content industry (819.231), Game industry (805.568), Korea (790.339), Assemblyman (749.254), Korea Association of Game Industry (689.621), COVID-19 (646.248), Amendment (544.657), Content (505.219), Ministry of Culture, Sports and Tourism (502.333), etc.

The degree of centrality shows the following: Game (0.148), Game industry (0.101), Content industry (0.095), Korea (0.062), COVID-19 (0.039), Content (0.034), Assemblyman (0.03), Ministry of Culture, Sports and Tourism (0.028), Korea Association of Game Industry (0.027), Global (0.026), etc.

In summary, it was found that Game, Game industry, Content industry, Assemblyman, and COVID-19 are the important keywords. The overall results are depicted in Table 4.

**Table 4:** The top 40 important keyword of game industry after COVID-19 outbreak in 2020.

Keyword	Freq	TF-IDF	Degree	Keyword	Freq	TF-IDF	Degree
Game	1232	1021.64	0.148	Game festival	144	434.183	0.013
Game industry	858	805.568	0.101	This year	130	350.124	0.015
Content industry	639	819.231	0.095	Game law	128	370.685	0.015
Korea	509	790.339	0.062	Government	122	353.309	0.015
Korea Association of Game Industry	344	689.621	0.027	Game business	102	297.708	0.014
Assemblyman	341	749.254	0.03	Propulsion	96	281.305	0.014
Covid-19	332	646.248	0.039	Development	96	287.055	0.012
Content	241	505.219	0.034	Business	92	277.395	0.01
Ministry of Culture, Sports and Tourism	235	502.333	0.028	Culture	91	273.235	0.015
Amendment	232	544.657	0.021	E-Sports	89	320.437	0.01
Hold	232	488.707	0.025	Future	88	266.455	0.01
Support	206	487.67	0.025	Actively	83	256.815	0.012
Announcement	196	449.009	0.025	Open	82	274.008	0.009
Online	188	443.817	0.024	G-Star	80	318.673	0.007
Global	182	433.281	0.026	Scale	79	251.214	0.011
Growth	180	421.413	0.026	Strategy	72	241.867	0.01
Promote	171	420.208	0.024	Change	72	230.037	0.009
Regulation	160	428.013	0.018	Digital	69	243.878	0.008
Export	149	421.779	0.02	Attention	68	218.295	0.011
Non-Contact	148	397.251	0.022	Korea Game Society	66	224.111	0.009

### CONCOR analysis of game industry in 2020

**Fig 9:**
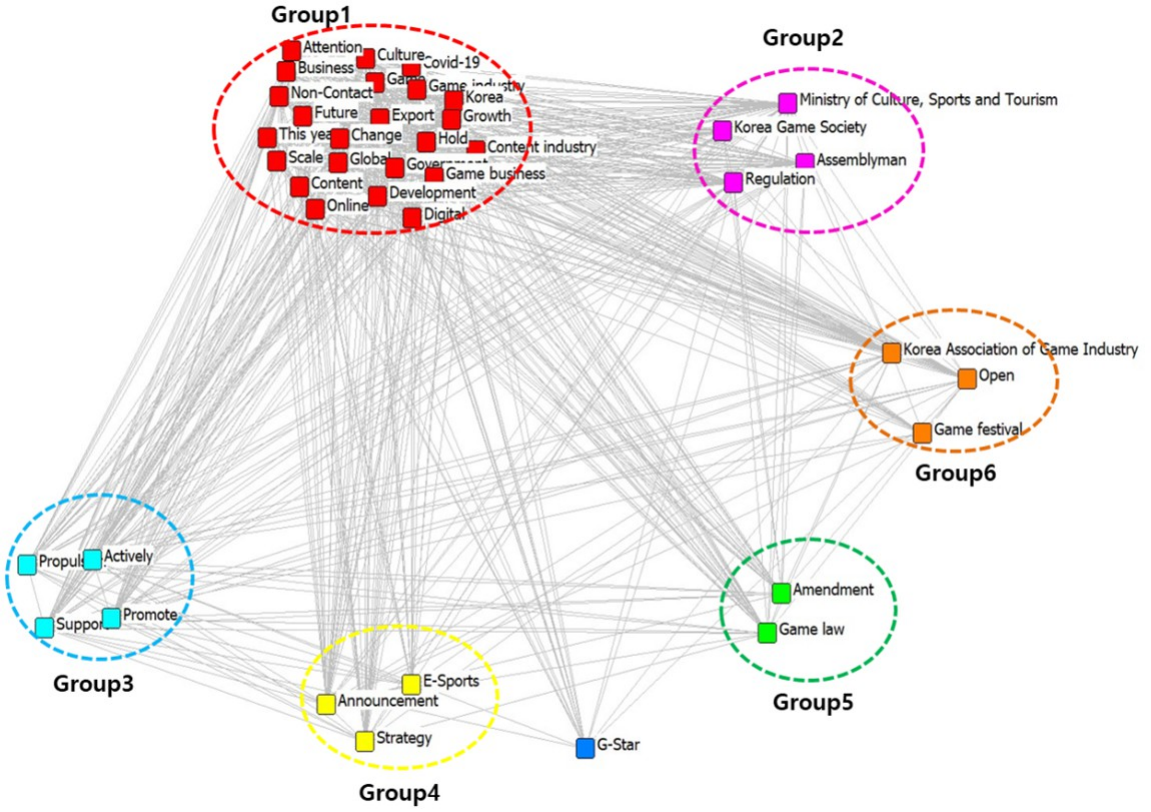
Visualization of CONCOR analysis of 40 important keywords in 2020.

By using the CONCOR analysis and UCINET6 program, its visualization is illustrated in Fig 9.

As a result of CONCOR analysis, Group 1 (red circle) includes Game, Game industry, Content industry, Korea, Game business, and Growth, thus, it is named ‘Game industry’. Group 2 (pink circle) includes Assemblyman, Regulation, Ministry of Culture, Sports and Tourism, thus, it is named ‘Game regulations’. Group 3 (sky blue circle) includes Policy enforcement and Support, thus, it is named ‘Industrial expansion’. Group 4 (yellow circle) includes Announcement, Strategy, and E-sports, thus, it is named ‘E-sports’. Group 5 (green circle) includes Game law and Amendment, thus, it is named ‘Game law amendment’. Group 6 (orange color circle) includes game festival and open, thus, it is named ‘Game festival’. The details are depicted in Table 5.

**Table 5:** The top 40 important keyword of game industry in 2020.

Group	Group name	Keyword
Group 1 (red circle) (23)	Game industry	Game, Game industry, Content industry, Korea, Game business, Growth, COVID-19, Content, culture, Non-Contact, Hold, This year, Business, Online, Global, Future, Change, Attention, Export, Development, Scale, Digital, Government
Group 2 (pink circle) (4)	Game regulations	Assemblyman, Regulation, Ministry of Culture, Sports and Tourism, Korea Game Society
Group 3 (sky blue circle) (4)	Industrial expansion	Propulsion, Support, Actively, Promote
Group 4 (yellow circle) (3)	E-sports	Announcement, Strategy, E-Sports
Group 5 (green circle) (2)	Game law amendment	Game law, Amendment
Group 6 (orange color circle) (3)	Game Festival	Game festival, Open, Korea Association of Game Industry

The keywords in 2020 can be classified into a total of six groups such as Game industry, Game regulations, Industrial expansion, E-sports, Game law amendment, and Game Festival. Those groups indicate that the national game industry of 2020 in South Korea continued to be in a growth trend and the Korean government planned to reduce the regulatory impediments to the game industry and rigid game rating system. The changes included changing the market policies, online game shutdown policy, lasting from 12:00 to 6:00 am, as a means of preventing game addiction in adolescents aged 15 or below, and investment in e-sports and online game infrastructure, eventually in order to expand the size of the game industry itself.

### Comparative analysis of game industry issues between 2019 and 2020

To clearly see the impact of the COVID 19 pandemic on online game issues in South Korea, the keywords between 2019 and 2020 were compared as indicated in Table 6.

**Table 6:** Game industry keywords comparison between 2019 and 2020.

Repeated Keywords	Actively, Assemblyman, Content, Content industry, Culture, Development, Export, Future, Game, Game business, Game industry, Global, Government, Growth, G-Star, Hold, Korea, Korea Association of Game Industry, Ministry of Culture, Sports and Tourism, Promote, Regulation, This year		
New Keywords (18)			
2019		2020	
Keywords	Frequency	Keywords	Frequency
Disease code	258	Covid-19	332
China	209	Amendment	232
Game addiction	184	Support	206
Investment	127	Announcement	196
Introduction	103	Online	188
Export amount	82	Non-Contact	148
Policy	80	Game festival	144
WHO	77	Game law	128
Copyright	76	Propulsion	96
Education	72	Business	92
Game disorder	72	E-Sports	89
Game institute	71	Open	82
Classification	68	Scale	79
Game awards	66	Change	72
Problem	66	Strategy	72
Field	62	Digital	69
Concern	61	Attention	68
Host	60	Korea Game Society	66

In the list of 2019 keywords, the words such as Disease code, Game addiction, and WHO (World Health Organization) were placed on the top 10 and their frequency was relatively higher than 70. These negative terms arose in association with the WHO declaration that gaming addiction would be listed as a mental health condition for the first time in May 2019. In contrast, the top-listed keywords of 2020 were contact-free, online, and digital, which indicates that the online game and its users had been greatly influenced by the COVID 19 pandemic, and what should be noticed was that the governmental support for the game law and E-sports market was much more being enforced.

## Discussion

In comparing the game industry issues between 2019 and 2020 based on the big data analysis of news data, the following implications were identified. Firstly, there was a great change in attitudes toward online gaming in the public and the government policies. When the world-renown health organization WHO designated and conditioned abnormal gaming behaviours as a mental illness, gaming became more associated with public health issues and family social problems. Thus, the game industry was also got negatively affected and the additional regulatory policies were enforced by the government.

However, after the COVID 19 outbreak, the restrictions and worries suddenly decreased but issues revolving around economic investment and the profitable market in the game industry gained an attention. Compared the sudden change in attitude and policies, the government should be careful not to be polarized in terms of public opinions and the policy-making towards the gaming and game industry. Rather, it is necessary to have in-depth discussions with business enterprises and academic circles regularly so that the overall content industry including the game industry can continue to grow in POST-COVID era.

Secondly, relevant government agencies need to make a sustained effort to maintain the growth trend in the game industry. The video games industry is immensely large and related to several industries such as computing, arts, music, and story content and experts to make it keep on. Thus, it requires newly trained game-related designers, programmers, technicians, artists, and researchers. In this regard, for the sustainable development of the domestic game industry, laws and regulations related to the game industry need to be reliable and planned on a long-term base.

Accordingly, it is necessary to build an industrial ecology through which business enterprises can grow by constructing cluster complexes specializing in developing startups and small and medium-sized businesses along with political support.

## Conclusion

The game industry has continued to grow and its growth rate increases more rapidly due to the great influence of COVID-19 on the person’s lifestyle and entertainment activities. This is a general trend in the overall content industry including animation, broadcasting, and online education. The comparative analysis between the ongoing game industry issues in 2019 and 2020 has indicated that there were significant changes in the society’s attitude towards gaming. That is, in 2019, negative keywords arose including disease code and game addiction as associated with the WHO declaration to include gaming addiction to mental illness. In 2020, during the COVID-19 pandemic, the top keywords that arose includes contact-free, online, and digital, which signifies that the game industry was influenced by the pandemic, and users tend to move towards online gaming. These results are significantly important to understand the ongoing trend in the gaming industry, to guide the establishment of public game policies, and predict future gaming trends for the better improvement of the entire game industry in South Korea.

The limitations in this paper have two aspects. First, one-year difference in this study is quite short to make a general conclusion about how the COVID-19 pandemic has influenced on the entire game industry in South Korea so further study will be necessary to conduct with an expanded data collection period and amount. Second, recent studies use various text mining techniques besides CONCOR or SNA that this paper applied. Thus, it requires to apply different methodology of text mining such as Topic Modeling, which is a machine learning technique that is capable of discovering the patterns within a set of documents, to check the analysis results to deduce more accurate and different aspects of meanings associated with the relationship between Covid-19 pandemic and game industry.
